# Distinct Circulating Biomarker Profiles Associated with Type 2 Diabetes in a Regional Cohort—A Cross-Sectional Study

**DOI:** 10.3390/metabo15120776

**Published:** 2025-11-30

**Authors:** Abdullah Alsrhani, Muhammad Atif, Aisha Farhana

**Affiliations:** Department of Clinical Laboratory Sciences, College of Applied Medical Sciences, Jouf University, Sakaka 72388, Saudi Arabia

**Keywords:** type 2 diabetes mellitus, biomarkers, vitamin D deficiency, hyperuricemia, risk association, Saudi Arabia, cross-sectional study, metabolic biomarker profiling

## Abstract

**Background:** The prevalence of Type 2 diabetes mellitus (T2DM) has significantly increased in Saudi Arabia, rising from 16.8% in 2018 to 28% in 2023. This study aimed to identify and quantify the key biochemical factors associated with T2DM patients in the Aljouf region by comparing a comprehensive panel of biomarkers between T2DM and healthy individuals and to identify key risk factors in the regional population. **Materials and Methods:** A cross-sectional study was conducted by enrolling 114 T2DM patients and 91 healthy controls recruited from tertiary care centers in the Aljouf region, Saudi Arabia. We analyzed lipid profiles, vitamin levels (B_12_ and D), liver profile, and renal function markers. Statistical analyses included independent *t*-test, Pearson’s correlation, and binary logistic regression to calculate the Odds Ratio (OR) with 95% confidence intervals (CI). **Results:** T2DM patients showed significantly altered metabolic profiles including elevated triglycerides (*p* = 0.041), LDL cholesterol (*p* = 0.017), and serum uric acid (*p* < 0.001) in addition to deficient levels of vitamin B_12_ (*p* < 0.001) and vitamin D (*p* < 0.001). In logistic regression analysis, hyperuricemia (OR = 4.85, 95% CI: 2.45–9.61) and vitamin D deficiency (OR = 3.62, 95% CI: 1.98–6.62) were the strongest independent predictors of T2DM, after adjusting for potential confounders. **Conclusions:** Our findings confirm a distinct biochemical phenotype in T2DM patients from the Aljouf region. The potent associations of hyperuricemia and vitamin D deficiency with T2DM suggest their utility as key biomarkers providing a regional context. These findings highlight their potential relevance for prioritizing public health and clinical interventions in the studied population.

## 1. Introduction

Diabetes mellitus (DM) is a predominant metabolic disorder characterized by chronic hyperglycemia resulting from defects in insulin secretion. The prevalence of T2DM has remarkably increased in the last decade, showing an upward trend from 16.8% in 2018 to 28% in 2023 in Saudi Arabia, positioning it as a national health crisis [1]. The World Health Organization projects that DM will rank as the seventh leading cause of mortality by 2030 [2]. It is widely recognized as a significant public health issue due to its high prevalence, the significant impact it has on society, and the potential for complications [3]. Chronic complications of DM can lead to conditions such dyslipidemia and proteinuria, which can hinder growth and contribute to degenerative complications over time [4]. It is worth noting that individuals with DM often experience additional health problems such as obesity, hypertension, microangiopathy, and metabolic syndrome.

This complexity has driven extensive scientific research aimed at understanding the intricate details surrounding DM. The goal of this research is to uncover the various aspects that play a significant part in the onset and progression of this disease. In doing so, they hope to create tailored treatment approaches that cater to the unique needs of each patient. Numerous studies have investigated the various factors, including genetics, environment, and lifestyle, that contribute to the development of T2DM [5].

A critical strategy in managing this complexity is patient profiling to successfully identify groups of patients who share similar characteristics and experience a similar progression in their illness. Profiling patients in this way has paved the way for targeted treatment interventions that can potentially benefit these specific patient subgroups. Within the field of health care, patient profiling has been utilized for a range of purposes. These include categorizing patients based on their risk of developing different diseases, predicting prognosis and disease advancement, customizing medical treatments, and identifying patient needs [6]. This demonstrates the power of a multi-factorial approach for complex diseases like T2DM, which is precisely the approach we adopt in this study by analyzing a comprehensive panel of biomarkers.

Understanding risk variables in patients with DM can help doctors create customized treatment strategies and interventions to lower the chances of complications and enhance patient outcomes. Certain biomarkers, such as high-sensitivity C-reactive protein (hs-CRP) and Hemoglobin A1c (HbA1c), also called glycated hemoglobin, have demonstrated their ability to forecast cardiovascular diseases (CVDs) in people with DM [7]. Albuminuria has been found to be a significant indicator of CVD in people with DM [8]. Medical researchers have discovered that biomarkers like B-type natriuretic peptide (BNP) and N-terminal pro-B-type natriuretic peptide (NT-proBNP) can be used to predict CVD in patients with DM, specifically heart failure. These natriuretic peptides have shown promising results in their ability to provide valuable insights into the health of patients [9]. Together, these biomarkers provide a clear picture of patient’s cardiovascular risk, in addition to blood sugar to include markers of inflammation, kidney function, and heart health, which is essential for creating better treatment plans.

Understanding the trajectories of body mass index (BMI) and waist circumference is crucial as they play an important part in the progression of T2DM and can be corrected to reduce the risk. There are several factors that are related to T2DM, such as blood pressure, glucose and lipid levels, renal function tests, β-cell function, insulin resistance, steatosis, and indicators of chronic inflammation [10]. Numerous studies have documented the progressive alterations in the fasting glucose, oral glucose tolerance test, β-cell function, and insulin resistance long before the onset of T2DM, with more pronounced and unfavorable changes occurring 3–5 years prior to diagnosis. There have been some studies conducted on the progressive alterations in other risk variables, like BMI, but the results have been inconclusive so far. This underscores that while we understand the metabolic patterns that lead to T2DM, a clear picture of how key anthropometric measures like BMI evolve during disease progress is still missing.

Given the vital role of vitamin B_12_ in various important bodily functions and the potential consequences of its deficiency, it is advised by the American Diabetic Association (ADA) to regularly check vitamin B_12_ levels in DM patients. This is particularly important for patients with anemia or peripheral neuropathy, which highlights the clinical importance of vitamin status in diabetes management [11]. Additionally, other vitamins, such as vitamin D, may also play a significant role. Several studies have described a link between insufficient vitamin D levels and a higher likelihood of experiencing CVD [12,13,14]. However, the precise mechanisms that connect the two have not yet been fully understood. Inflammation has been widely documented as a key factor in the onset and progression of atherosclerosis [15]. Past observational findings have indicated a potential link between insufficient levels of vitamin D and raised levels of CRP and other inflammatory cytokines in the bloodstream [16]. Meanwhile, previous studies have indicated that supplementation with vitamin D can help improve the overall inflammatory profile in T2DM patients [17]. Hence, a panel of biomarkers, including vitamins and other circulating factors, may offer a more holistic view of an individual’s metabolic risk profile.

Despite this knowledge, a significant gap remains. While the roles of individual biomarkers like cholesterol and vitamin D are well-known globally, we still lack a clear picture of how a wide range of these biomarkers including lipids, vitamins, liver profile, and kidney function markers collectively relate to T2DM in the Saudi population. Our study sought to fill this gap. We conducted a detailed comparison of the biochemical profiles of T2DM patients and healthy individuals in the Aljouf region. Our main goals were to pinpoint which biomarkers are most significantly altered and to create a clear association with T2DM status. Our findings will lay a practical framework for the region to better assess T2DM status and develop more personalized strategies for patients.

## 2. Material and Methods

### 2.1. Study Population

A cross-sectional, case–control study was conducted in June 2023–April 2024. Participants of both sexes aged over 20 years were recruited in this study. Healthy people without acute or chronic illness were randomly selected as healthy controls. T2DM patients were enrolled from the Endocrinology and Diabetes Centre at King Abdul Aziz Specialty Hospital and Prince Mutaib Hospital, Sakaka, Aljouf region. T2DM in patients was confirmed according to the guidelines of ADA and patients’ blood test for fasting blood glucose. Blood samples were obtained from 205 individuals (91 males and 114 females), categorized into non-diabetic healthy individuals and with T2DM. The sample size was calculated using a power analysis conducted with G*Power 3.1 software (G*Power software, version 3.1.9.7; Franz Faul, Universität Kiel, Kiel, Germany). This analysis indicated a minimum required sample size of 196 participants. Our final analytical cohort of 205 participants, therefore, exceeds this requirement.

To confirm the achieved statistical power of our study, a post hoc power analysis was performed using the same software (G*Power software, version 3.1.9.7; Franz Faul, Universität Kiel, Kiel, Germany). This analysis calculated the observed statistical power based on the final sample size (N = 205), the pre-specified alpha level (α = 0.05), and the effect sizes measured with Odds Ratios obtained from the final multivariate logistic regression model for the significant predictors. This analysis confirmed that the study achieved a statistical power exceeding 99% for detecting the effects of the key predictors.

A total of 280 potential participants were initially screened for this study. To ensure a homogeneous cohort and minimize confounding factors, we applied strict exclusion criteria. Specifically, we excluded 75 patients due to pre-existing renal impairment, use of medications affecting uric acid/vitamin levels, the presence of other endocrine disorders, pregnant and lactating women, persons with a history of gout or incomplete laboratory data. Also, participants with hepatic, kidney, and CVD were also excluded from the study. This process yielded a final analytic cohort of 205 patients, ensuring a high-quality dataset for robust statistical analysis

### 2.2. Sociodemographic Data Collection

Data collected included the general health status of each participant, anthropometric characteristics (such as sex age, height, and weight), medication use, and family disease history (Table 1). A measuring tape was used to measure the body height up to the closest 0.1 cm, and weight was recorded to the nearest 0.1 kg with a digital weighing scale (MOMAX smart, lite tracker, MOMAX, Hong Kong, China.) without shoes and with light clothes. Body weight (kg) was divided by body height (m^2^) to calculate the BMI (kg/m^2^). All measurements were performed in replicates to ensure the accuracy of the data. The T2DM patients were in early- to mid-stage disease according to the clinical assessment and patients’ history.

### 2.3. Blood Sampling and Laboratory Testing

Venipuncture was performed to collect blood samples from all participants. To ensure analyte stability, the samples were immediately placed in an icebox and transferred to the medical laboratory for testing. All blood samples were processed within one hour of collection. Specimens were centrifuged for 15 min at 3000 rpm to isolate the serum. Subsequently, the serum was preserved at −20 °C until testing was carried out. All biochemical parameters were analyzed in the same certified clinical laboratory. Lipid profile, liver profile, and renal function tests were performed using the colorimetric method on a chemistry analyzer (Mindray BS300), and vitamin D and vitamin B_12_ levels were determined using the ELISA technique on an automated ELISA machine (Human Elisys Quattro automated analyzer). All laboratory analyses were performed following strict standard operating procedures and using manufacturer-provided calibrators and controls. Intra-assay and inter-assay coefficients of variation (CV) for all assays were maintained below 5%, ensuring analytical precision.

### 2.4. Diabetes Diagnostic Criteria

The ADA criterion was followed, confirming DM when a fasting plasma glucose level ≥126 mg/dL and a non-fasting plasma glucose concentration ≥200 mg/dL were observed. Self-reported insulin or hypoglycemic medication use in recent days was also considered a confirmation of DM. Non-diabetic healthy participants (normoglycemia) were identified as having a fasting plasma glucose concentration of  <100 mg/dL and lack the criteria that define pre-diabetes or DM. Inclusion criteria: Patients with confirmed T2DM were entered into the study regardless of age, sex, and BMI. Exclusion criteria: Pregnant and lactating women were excluded, as well as any seriously ill patient with alterations in sensory and higher functions. Patients suffering from acute heart, renal and liver disease, infections, cancer, or critical illness were excluded from the study.

### 2.5. Statistical Analyses

All statistical analyses were performed using IBM SPSS Statistics (Version 24). Continuous variables, including demographic and biochemical data, are presented as mean ± standard deviation and were compared between the T2DM and control groups using independent sample *t*-tests. To ensure the robustness of these comparisons against potential confounding, an Analysis of Covariance (ANCOVA) was employed for key biochemical parameters, with age included as a covariate. Categorical data, such as family history and sex, are presented as frequencies and percentages, and group differences for these variables were evaluated using the Chi-squared test. For all analyses, a two-tailed *p*-value of less than 0.05 was considered statistically significant. To quantify the strength of association between the significantly altered biochemical parameters and T2DM status, univariate logistic regression was performed to calculate Odds Ratios (OR) with 95% confidence intervals (CI).

### 2.6. Ethical Approval

Ethical approval was acquired from the Local Committee of Bioethics of Jouf University (Approval Number: 7-11-44). The study methods were conducted purely in compliance with the standard guidelines and approved protocols.

### 2.7. Patient Consent Statement

Before inclusion in the study, written informed consent was taken from all individuals included in the study.

## 3. Results

### 3.1. Study Population Characteristics

A total of 205 participants were included in the final analysis, comprising 114 patients with Type 2 diabetes mellitus (T2DM) and 91 healthy controls. The demographic and clinical characteristics are summarized in Table 1. The two groups were well-matched for most baseline characteristics, with no statistically significant differences in age (*p* = 0.147), sex distribution (*p* = 0.478), blood pressure, height, weight, or BMI (all *p* > 0.05). As expected, the T2DM group had a significantly higher prevalence of family history of diabetes (*p* < 0.001) and markedly elevated HbA1c levels (*p* < 0.001), confirming the glycemic distinction between the groups.

### 3.2. Alterations in Lipid and Vitamin Profiles

**Figure 1:**
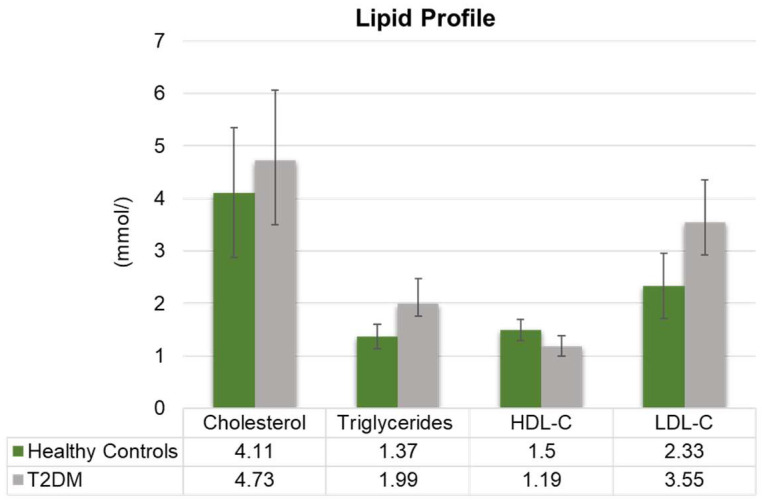
Lipid profile of T2DM patients (gray) compared to healthy controls (green). The profile includes cholesterol, triglycerides, HDL-C, and LDL-C). The graph shows a significant rise in the TG (*p* = 0.041) and LDL-C (*p* = 0.017) serum levels compared to those of the control group. The *p*-values for cholesterol and HDL-C were non-significant. The critical clinical thresholds for the assessed parameters were the following: LDL-C, Optimal (<2.6 mmol/L) and High (≥4.1 mmol/L); triglycerides, Normal (<1.7 mmol/L) and High (≥2.3 mmol/L); for HDL-C, Low (<1.0 mmol/L) and Protective (≥1.6 mmol/L); and total cholesterol, Desirable (<5.2 mmol/L) and High (≥6.2 mmol/L).

**Figure 2:**
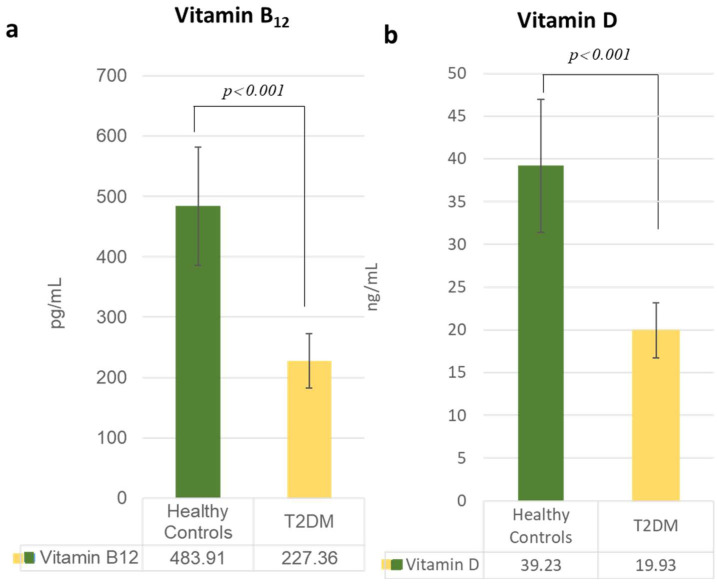
Vitamin B_12_ and vitamin D profiles of T2DM (yellow) compared to healthy control (green). The serum levels of (**a**) vitamin B_12_ and (**b**) vitamin D are observed to be significantly lower (*p* < 0.001) in patients with T2DM. The clinical reference ranges are as follows: Vitamin B_12_: Deficient (<200 pg/mL), Borderline (200–300 pg/mL), Normal (>300 pg/mL). Vitamin D: Deficient (<12 ng/mL), Insufficient (12–20 ng/mL), Sufficient (≥20 ng/mL).

Analysis of serum biomarkers revealed significant differences between T2DM patients and healthy controls. The lipid profile was notably atherogenic in the T2DM cohort, with significantly elevated levels of triglycerides (*p* = 0.043) and LDL-C (*p* = 0.017), in addition to a decrease in HDL-C (Figure 1). Furthermore, circulating levels of Vitamin B_12_ and Vitamin D were substantially lower in T2DM patients compared to controls (*p* < 0.001) (Figure 2).

### 3.3. Liver Profile and Kidney Function Profiles

**Figure 3:**
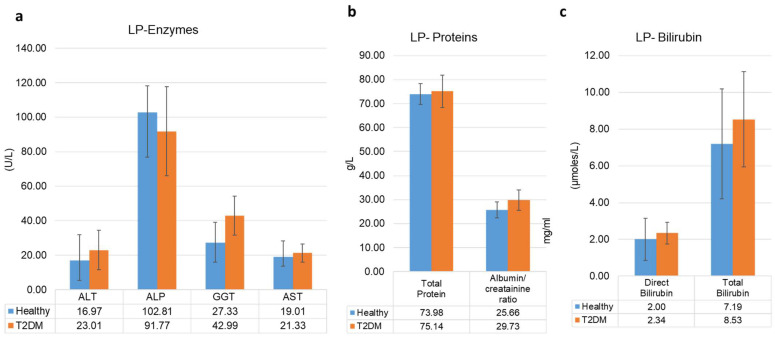
Liver profile (LP) of T2DM patients (orange) compared to healthy controls (blue). The profile includes (**a**) enzymes (ALT, ALP, GGT and AST), (**b**) proteins (total and albumin, used as the albumin to creatinine ratio—ACR), and (**c**) bilirubin (direct and total bilirubin). Overall, the graph does not indicate a significant rise in any of the liver profile markers. Hence, the *p*-values for other LFT parameters were non-significant. The clinical reference values of the assessed biomarkers were as follows: Enzymes (U/L): ALT (M: 10–40, F: 7–35), AST (5–40), ALP (40–130), GGT (M: 8–61, F: 5–36). Proteins (g/L): total protein (64–83), ACR (<30 mg/g). Bilirubin (μmol/L): Total (<21), Direct (<5).

**Figure 4:**
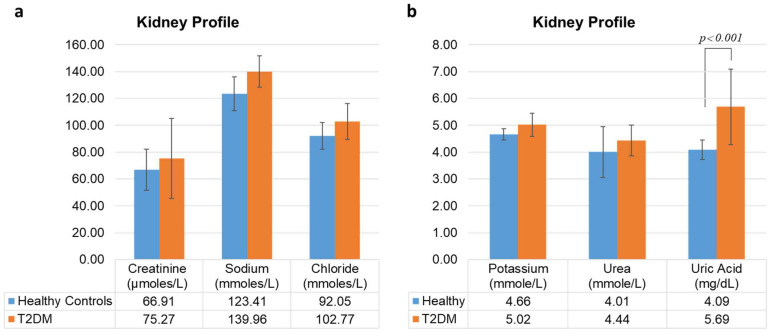
Kidney function profile of T2DM patients (orange) compared to healthy controls (blue). The serum levels of metabolites include metabolites (creatinine, urea, and uric acid) and ions (sodium, chloride, magnesium, and potassium). (**a**) shows the serum levels of metabolites (creatinine, urea, and uric acid), while (**b**) shows the serum levels of ions (sodium, chloride, magnesium, and potassium). Overall, the graph indicates a significant rise (*p* < 0.001) only in the serum levels of uric acid compared to the control group. The *p*-values for other KFT parameters were non-significant. Normal reference ranges for each parameter: creatinine (62–115 µmol/L), urea (2.5–7.1 mmol/L), uric acid (3.4–7.2 mg/dL), sodium (135–145 mmol/L), chloride (92–107 mmol/L), magnesium (0.70–1.05 mmol/L), and potassium (3.5–5.3 mmol/L). Statistical analysis indicates a significant increase (*p* < 0.001) in the serum levels of uric acid in the T2DM cohort. The *p*-values for all other measured parameters were non-significant.

The liver profile tests comprise proteins (total protein and albumin, assessed through the albumin to creatinine ratio, ACR), bilirubin, enzymes (alanine transaminase (ALT), alkaline phosphatase (ALP), gamma-glutamyl transferase (GGT), and aspartate transaminase (AST). Analysis revealed a preserved hepatic profile in the T2DM cohort (Figure 3). The measured parameters, including liver enzymes (ALT, ALP, GGT, AST), proteins (total protein), bilirubin (direct and total), and the albumin to creatinine ratio (ACR), showed no statistically significant differences between the T2DM patients and the healthy control group. These findings indicate an absence of significant hepatocellular injury, cholestasis, or synthetic dysfunction in the studied T2DM population. The renal function profile, comprising metabolites (creatinine, urea, uric acid) and serum electrolytes (sodium, chloride, magnesium, potassium) also showed a distinct pattern (Figure 4). A highly statistically significant elevation in serum uric acid was observed in the T2DM group compared to healthy controls (*p* < 0.001). In contrast, the levels of other renal metabolites, including creatinine and urea, as well as all measured electrolytes, did not differ significantly between the groups. This finding suggests that hyperuricemia may serve as an early and prominent indicator of metabolic renal dysfunction in individuals with T2DM, preceding overt alterations in other conventional markers of kidney function.

### 3.4. Multivariate Logistic Regression Analysis of Biomarker Associations with T2DM

To identify independent predictors of T2DM, a multivariate logistic regression analysis was performed, adjusting for potential confounders. The analysis revealed several biochemical parameters significantly associated with T2DM status, as detailed in Table 2. High serum uric acid demonstrated the strongest association, emerging as the most potent independent predictor with an OR of 3.39 (95% CI: 1.33–8.62, *p* = 0.010). This indicates that the odds of having T2DM increased by over three-fold. Vitamin D and vitamin B_12_ also showed significant associations with T2DM. Lower levels of vitamin D were associated with a 2.77-fold increase in the odds of T2DM (95% CI: 1.50–5.11, *p* = 0.001), while reduced vitamin B_12_ levels conferred a 2.26-fold increase in odds (95% CI: 1.38–3.70, *p* = 0.001). Among lipid parameters, both TG and LDL-C were significant independent predictors. Elevated TG levels were associated with a 2.31-fold increase in T2DM odds (95% CI: 1.05–5.09, *p* = 0.041), and higher LDL-C levels with a 1.95-fold increase (95% CI: 1.13–3.37, *p* = 0.017).

### 3.5. Pearson’s Correlation Analysis of T2DM Related Biomarkers

**Figure 5:**
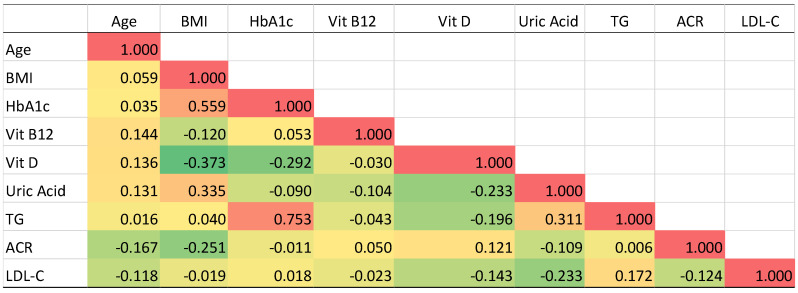
Correlation matrix of clinical parameters in the study population. The heatmap displays Pearson’s correlation coefficients between nine clinical variables: age, BMI, HbA1c, vitamin B_12,_ vitamin D, uric acid, triglycerides, ACR, and LDL-C. Correlation coefficients range from −1 to +1. The color gradient represents the strength and direction of each association: strong positive correlations are shown in deep shades of red, weaker positive correlations transition to lighter reds and yellow, while negative correlations are represented by a gradient of green, with deeper green indicating stronger inverse relationships. Coefficients near zero (indicating no linear association) appear in pale yellow. Key observations include a strong positive correlation between HbA1c and TG (r = 0.753), a moderate positive correlation between BMI and HbA1c (r = 0.559), and a weak-to-moderate positive correlation between BMI and uric acid (r = 0.335). Notable negative correlations include vitamin D with both BMI (r = −0.373) and HbA1c (r = −0.292). Most other correlations were weak or negligible.

Pearson’s correlation analysis was conducted to examine the interrelationships between key clinical and biochemical parameters in the study population. The resulting correlation matrix is presented in Figure 5.

A strong positive correlation was observed between HbA1c and triglycerides (TG) (r = 0.753). HbA1c also demonstrated a moderate positive correlation with body mass index (BMI) (r = 0.559). In contrast, HbA1c showed weak negative correlations with vitamin D (r = −0.292) and uric acid (r = −0.090). BMI exhibited a moderate positive correlation with uric acid (r = 0.335) and a moderate negative correlation with vitamin D (r = −0.373). Vitamin D levels were inversely correlated with both HbA1c (r = −0.292) and BMI (r = −0.373). Vitamin B12 showed minimal correlation with other parameters, with all coefficients below 0.15. Notably, LDL-C demonstrated weak correlations with all other parameters (r < 0.24), with the strongest being a weak positive correlation with TG (r = 0.172) and a weak negative correlation with uric acid (r = −0.233). ACR levels showed minimal association with other variables, with correlation coefficients ranging from −0.251 to 0.121. Age demonstrated generally weak correlations with all other parameters (r < 0.17), with the strongest being weak positive correlations with vitamin B12 (r = 0.144) and vitamin D (r = 0.136).

Overall, the findings of this study shed light on the altered biochemical parameters in T2DM patients and highlight the necessity of implementing focused treatments to control hyperuricemia, vitamin deficiencies, and other high-risk variables in order to slow the course of the illness.

## 4. Discussion

Our analysis of a regional Saudi cohort revealed a distinct biochemical profile in patients with T2DM, characterized by atherogenic dyslipidemia, hypovitaminosis D and B_12_, and hyperuricemia. Multivariate logistic regression identified hyperuricemia and vitamin D deficiency as the strongest independent predictors of T2DM, with Odds Ratios of 3.39 and 2.77, respectively. Furthermore, vitamin B_12_ deficiency, elevated TG, and high LDL-C were also significantly associated with the disease. Correlation analysis further elucidated key metabolic interrelationships, including a strong positive association between HbA1c and triglycerides (r = 0.753) and a moderate positive correlation between HbA1c and BMI (r = 0.559). Furthermore, vitamin D levels demonstrated inverse correlations with both HbA1c (r = −0.292) and BMI (r = −0.373), while most other inter-parameter correlations were weak or negligible.

The results of our study directly contribute to the established field of diabetology, where patient profiling is extensively pursued to stratify risk and personalize management strategies [18,19]. Sheehan et al. investigated profiling factors for DM patients that would facilitate more convenient and effective physician access [19]. Profiling was utilized by Li et al. to determine the hospitalization risk and mortality among DM patients [20]. Furthermore, Zghebi et al. devised a scoring system using information regularly gathered in the electronic health record to evaluate the likelihood of hospitalization and death in people with DM [21]. Our study extends this progress by delineating a distinct biochemical phenotype associated with T2DM in a regional cohort of Saudi Arabia.

The findings from our analysis of 205 participants reveal a pattern of metabolic dysregulation. The lipid profile of T2DM patients was significantly altered, marked by elevated levels of TG and LDL-C compared to healthy controls (Figure 1). This pattern of atherogenic dyslipidemia is a well-established factor of cardiovascular risk in T2DM (Figure 1). Our results align with global trends that reported higher total cholesterol and TG levels in DM patients in the Brazilian population [22]. Based on a study conducted by Parhofer and Laufs, TG levels exceeding 150 mg/dL can be categorized as moderate hypertriglyceridemia, which correlates with an elevated risk of CVD. On the other hand, levels surpassing 1000 mg/dL are classified as severe hypertriglyceridemia and greatly elevate the chances of developing acute pancreatitis [23]. This underscores the necessity of proper lipid management in our regional T2DM population to mitigate these associated risks.

Simultaneously, our study also identified a deficiency in vitamin D and vitamin B_12_ in T2DM patients, consistent with the previous studies. Gao et al., found the prevalence of vitamin B_12_ insufficiency at (2.15%) and borderline deficiency at (13.66%), while Aroda et al. and Reinslatler et al. reported insufficiency (4.3% and 5.8%) and deficiency (19.1% and 16.2%), respectively [24,25]. The implications of vitamin D deficiency are not specific to bone health but play a critical role in modulating cardiovascular risk. Talmor Y et al. found that vitamin D helps mitigate the detrimental effects of advanced glycation end products in cultured endothelial cells; its deficiency contributes to the onset of atherosclerosis by influencing inflammatory cytokines and directly impacting the blood vessels [26]. According to Dey et al., it was found that there is a relation between vitamin D deficiency, inflammatory cytokines, and risk for CVD [14]. This interplay highlights the need for a comprehensive management approach that addresses both conventional lipid parameters and micronutrient status.

Our multivariate logistic regression model, adjusted for key confounders, identified elevated serum uric acid as the strongest independent predictor of T2DM (OR = 3.39). This finding aligns with a growing body of evidence linking hyperuricemia to insulin resistance and β-cell dysfunction [27]. Higher levels of uric acid have been found to contribute to cytokine secretion and have been linked to endothelial dysfunction and systemic inflammation. The pro-inflammatory and pro-oxidant state induced by high uric acid levels, particularly within the context of metabolic syndrome, is hypothesized to contribute to endothelial dysfunction and systemic inflammation, thereby promoting the pathogenesis of T2DM [28,29]. Multiple investigations have documented a positive correlation between elevated levels of serum uric acid and the development of DM [30,31,32]. While some studies have reported conflicting results, our robust Odds Ratio, derived from a well-phenotyped cohort, strongly supports a significant role for uric acid in the regional manifestation of T2DM [33].

The strong association between hyperuricemia and T2DM identified in our cohort needs an understanding within the broader context of interconnected metabolic disorders, particularly the triangular relationship among Metabolic Dysfunction-Associated Steatotic Liver Disease (MASLD), elevated uric acid, and T2DM [34]. This triad likely operates through a self-perpetuating cycle of insulin resistance, systemic inflammation, and oxidative stress, with recent evidence strengthening these mechanistic links. Hyperuricemia is increasingly considered a potential contributor to both MASLD and T2DM pathogenesis. Elevated serum uric acid can directly induce endothelial dysfunction and stimulate the production of pro-inflammatory cytokines such as TNF-α and IL-1β [29]. Furthermore, uric acid generation is intrinsically linked to fructose metabolism. High fructose intake, a known driver of MASLD, accelerates ATP degradation in the liver, leading to increased uric acid production. This same pathway promotes de novo lipogenesis, thereby exacerbating hepatic steatosis [35,36]. The state of chronic, low-grade inflammation and hepatic insulin resistance inherent in MASLD impairs insulin clearance, contributing to systemic hyperinsulinemia [37]. This hyperinsulinemia, in turn, promotes renal sodium and urate reabsorption, thereby elevating serum uric acid levels and creating a vicious cycle. Simultaneously, hepatic steatosis and inflammation drive gluconeogenesis and worsen overall glycemic control, directly stimulating the progression of T2DM [38].

In our study, hyperuricemia was the strongest independent predictor of T2DM, even without alterations in other liver enzymes. This raises the possibility that uric acid may be an early, sensitive marker of underlying metabolic disturbances, such as subclinical MASLD, which precedes the full manifestation of blood glucose dysregulation. A study confirmed that individuals with MASLD have a significantly higher risk of developing hyperuricemia, and vice versa, reinforcing their bidirectional relationship [39]. Simultaneously, the pro-inflammatory milieu of MASLD, characterized by elevated cytokines like IL-6 and TNF-α, further aggravates peripheral insulin resistance and pancreatic beta-cell dysfunction, directly fueling the progression of T2DM.

Therefore, the identification of hyperuricemia in an individual should prompt a complete metabolic evaluation, including screening for both T2DM and MASLD. Future longitudinal studies in our population are warranted to evaluate the temporal sequence within this triad, which will be crucial for developing targeted interventions aimed at breaking this cycle and preventing the progression of metabolic disease.

Furthermore, contrary to the conventional view, our data revealed that lower levels of both vitamin D (OR = 2.77) and vitamin B_12_ (OR = 2.26) were associated with increased odds of T2DM. This association indicates a more complex pathophysiology in the disease process. For vitamin D, this may reflect a compensatory upregulation in response to underlying inflammatory processes or a disruption in its metabolic pathway akin to the vitamin D paradox observed in other inflammatory conditions [40]. The results for vitamin B_12_ are particularly intriguing and may be influenced by factors such as dietary patterns, supplement use, or altered cellular uptake in a diabetic state. These associations underscore the necessity of investigating the dynamic, and potentially multichannel roles of vitamins in advanced metabolic disease.

Pearson’s correlation matrix (Figure 5) provides insight into the interrelationships between key metabolic variables in our cohort. The observed moderate positive correlation between HbA1c and BMI (r = 0.559) confirms that adiposity remains a significant contributor to glycemic control in these established T2DM patients, consistent with the well-documented link between obesity and insulin resistance [41]. However, other factors, such as beta-cell exhaustion or medication effects, also significantly influence hyperglycemia. It is worth noting that the likelihood of developing DM rises steadily as BMI increases. For individuals who are overweight, the risk stands at 2%, while for those who are obese, the risk jumps to 21% [42]. Furthermore, the strong positive correlation between HbA1c and triglycerides (r = 0.753) reinforces a key axis of metabolic dysfunction, tightly linking poor glycemic control with diabetic dyslipidemia. Notably, the moderate negative correlation of vitamin D with HbA1c (r = −0.292) suggests a potential protective role or compensatory consumption, while its negative correlation with BMI (r = −0.373) points to a complex relationship with adiposity that warrants further investigation. Increased weight can result in insulin resistance and contribute to the onset of atherosclerosis through various mechanisms, such as the elevation of blood pressure and abnormal lipid levels [43]. Visceral obesity is linked to resistance to insulin actions and high levels of insulin, as well as issues like dyslipidemia, T2DM, hypertension, and increased risk for CVD.

**Figure 6:**
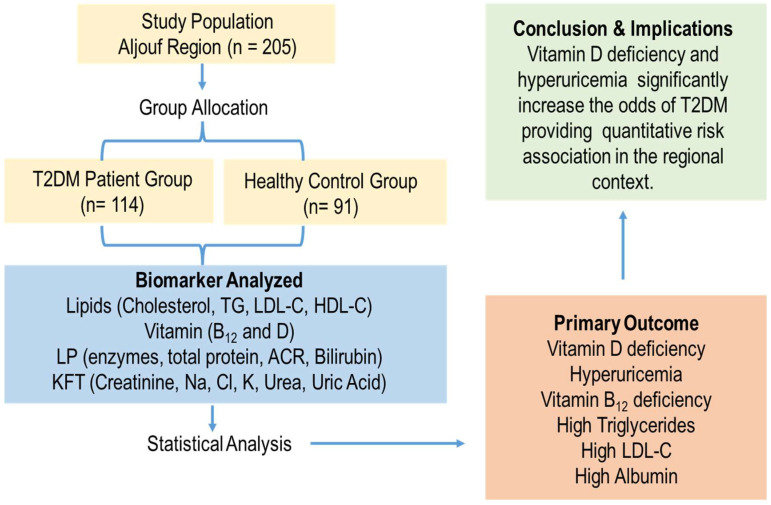
Study overview of biomarker associations with Type 2 diabetes mellitus (T2DM) in the study population. This study included 205 participants (114 T2DM patients, 91 healthy controls). Analyzed biomarkers included lipid profiles, vitamins (B_12_, D), liver profile (LP) tests, and kidney function tests (KFT). The primary outcome confirmed a significant association between T2DM and both vitamin D deficiency and hyperuricemia, providing a quantitative risk assessment for this population.

The findings from our cohort of 205 patients provide strong evidence for the involvement of non-traditional biomarkers in T2DM. In summary, our study delineates a distinct biochemical phenotype of T2DM in the Aljouf region, characterized by hyperuricemia, dyslipidemia, and association with vitamin status, particularly for vitamins D and B_12_ (Figure 6). We propose that integrating uric acid and vitamin status assessment into routine clinical profiling could significantly enhance preemptive health care strategies aimed at mitigating the growing burden of T2DM in Saudi Arabia. A potential consideration in measuring vitamin D is seasonal variation. However, our study design mitigates this concern, as sample collection spanned ten months (June 2023 to April 2024), capturing the full seasonal cycle of the Aljouf region. Consequently, our reported vitamin D levels are likely representative of the participants’ annual average exposure, strengthening the generalizability of its observed association with T2DM in this population.

Nonetheless, this study, primarily due to its cross-sectional nature, prevents any inference of causality between the observed biomarkers and T2DM, and the findings only represent association. Furthermore, while our multivariate model was adjusted for key biochemical parameters, the absence of data on specific medications (e.g., metformin, diuretics), detailed dietary habits, and physical activity levels means that residual confounding cannot be ruled out. However, the strong and significant associations identified provide a compelling rationale for future longitudinal studies to confirm the temporal sequence and explore the potential causative role of factors like hyperuricemia in the pathogenesis of T2DM.

## 5. Conclusions

This study identifies a distinct biochemical phenotype of T2DM in the Aljouf cohort, characterized by hyperuricemia, atherogenic dyslipidemia, and altered vitamin metabolism. Serum uric acid emerged as the strongest independent predictor, underscoring its potential role in T2DM pathogenesis. The identified associations and correlational patterns suggest that integrating uric acid and vitamin status into clinical profiles could enhance personalized management and preventive strategies for T2DM in this population. This study was designed to identify the core biochemical signature of T2DM, controlling for key metabolic interdependencies through multivariate logistic regression. Future research can now build upon this biochemical foundation by integrating data on medications, diet, and lifestyle to explore their specific modulating effects.

## Figures and Tables

**Table 1:** Demographic and anthropometric characteristics of study population.

	Healthy Controls	T2DM	*p*-Values
Number of participants	91	114	N/A
Age	47.26 ± 12.11	49.27 ± 9.11	0.147
Sex (M/F)	M (46); F (45)	M (51); F (58)	0.478
Nationality	Saudi	Saudi	N/A
Family History of Diabetes	Yes (10%); No (90%)	Yes (48%): No (52%)	<0.001 *
Family History of Thyroid Disease	No	No	N/A
Family History of Liver or Renal Disease	No	No	N/A
Blood Pressure Values Averages (Systolic)	125.58	126.98	0.387
Blood Pressure Values Averages (Diastolic)	80.59	82.20	0.192
Height (cm)	161.79 ± 12.44	164.20 ± 9.01	0.118
Weight (kg)	81.00 ± 14.72	81.56 ± 16.01	0.966
BMI	29.84 ± 3.41	30.11 ± 4.52	0.667
HbA1c (%)	4.89 ± 0.71	7.62 ± 0.82	<0.001 *
Medication	None (6 participants on occasional NSAID)	Insulin	N/A

* Statistical significance at *p* < 0.05.

**Table 2:** Association between serum biomarkers and T2DM (N = 205). Results from multivariate logistic regression analysis are shown. Odds Ratios (OR) are presented with their 95% confidence intervals and *p*-values.

Biochemical Parameters	Odds Ratio (OR)	95% CI	*p*-Value
Increased Uric Acid	3.39	1.33–8.62	0.010
Low Vitamin D	2.77	1.50–5.11	0.001
Low Vitamin B12	2.26	1.38–3.70	0.001
TG	2.31	1.05–5.09	0.049
LDL-C	1.95	1.13–3.37	0.017

## Data Availability

Data is contained within the article. Further inquiries can be directed to the corresponding author.
